# Integrated Application of GPR and Ultrasonic Testing in the Diagnostics of a Historical Floor

**DOI:** 10.3390/ma13112547

**Published:** 2020-06-03

**Authors:** Magdalena Rucka, Erwin Wojtczak, Monika Zielińska

**Affiliations:** 1 Department of Mechanics of Materials and Structures, Faculty of Civil and Environmental Engineering, Gdańsk University of Technology, Narutowicza 11/12, 80-233 Gdańsk, Poland; erwin.wojtczak@pg.edu.pl; 2 Department of Technical Fundamentals of Architectural Design, Faculty of Architecture, Gdańsk University of Technology, Narutowicza 11/12, 80-233 Gdańsk, Poland; monika.zielinska@pg.edu.pl

**Keywords:** non-destructive testing, historical floor, integrated diagnostics, ground penetrating radar, ultrasonic testing, in situ surveys, finite-difference time-domain modeling

## Abstract

The paper presents the results of integrated ground penetrating radar (GPR) and ultrasonic testing (UT) measurements conducted on a historical floor in St. Nicholas’ Church, Gdańsk, Poland. The described inspection was the first stage of the technical state assessment of the building. The aim of the study was the detection of underfloor air gaps, which were observed in a few trial pits. The condition of the ground under the floor was determined by localizing other inclusions such as rubble, human remains, brick walls and pipes. To identify the phenomenon of electromagnetic and ultrasonic wave propagation within the air gap, laboratory tests were conducted on physical models consisting of two concrete slabs stacked on top of each other and gradually moved apart to simulate a slot of varying thickness. The conducted research was supported by the numerical models of electromagnetic wave propagation. The obtained results showed that the integration of the GPR and UT methods provided an effective imaging of the floor and the area under it. Ultrasonic testing was proved to be a good technique for identifying air voids, while the GPR method allowed detecting concentrated anomalies and determining the degree of ground homogeneity under the floor.

## 1. Introduction

Non-destructive testing (NDT) is commonly used for assessing the condition of components of engineering structures. It is a quick and efficient approach, the main advantage of which is the ability to examine a structure in a non-invasive way, without damaging or changing the composition or shape of the inspected object. NDT covers a variety of techniques based on a wide range of physical phenomena, including propagation of elastic waves, being the basis for ultrasonic testing (UT), and electromagnetic waves used in the ground penetrating radar (GPR) method. Non-destructive testing can be applied on a selected part of a structure or for a comprehensive inspection of large-size objects, like bridges [1,2,3], water gates [4], retaining walls [5] or archaeological sites [6,7,8].

Non-destructive testing is particularly suitable in the case of historical objects. Such an approach is more and more often applied in cultural heritage buildings due to the necessity to preserve such structures in an untouched condition for future generations. The exact structure of historical objects dated several centuries back is usually unknown, since the technical documentation is incomplete or entirely gone. An efficient method to collect information in such situations is in situ inspection, often supported by numerical analyses that provide design guidelines and recommendations for planned reconstruction, strengthening and restoration works [9,10]. Non-invasive testing conducted within the flooring area allows detecting crypts, tombs and hidden rooms, as well as evaluating the technical condition of the floors and ceilings. GPR was successfully used for the diagnostics and condition assessment of different historical objects [11,12,13,14,15,16,17]. Despite the advances in non-destructive testing technology, there is no one technique suitable for every situation. Researches are often carried out using several methods to ensure that the obtained results are correct. Drahor et al. [18] compared the results obtained using the GPR and electrical resistivity tomography (ERT) while searching for cracks and damages that could occur in the church floor. Perez-Gracia et al. [19] compiled two methods, GPR and the capacitively coupled resistivity method, to obtain 2D images of the shallow subsurface under and around the Cathedral of Mallorca. The ground penetrating radar technique and laser scanning technique were combined by Tapete et al. [20] in order to interpret the displacements influencing the condition of the archeological monuments. Moropoulou et al. [21] presented the implementation of the integrated non-destructive methods, such as digital image processing, infrared thermography, ground penetrating radar, ultrasonic testing and fibre-optic microscopy for the inspection of historical objects. They detected air voids, delamination, moisture, material wear and degradation in the evaluation of the effectiveness of interventions and the assessment of the compliance of the repair method used. Faella et al. [22] took comprehensive measurements of a church in Bethlehem and estimated the condition of the floors, walls and columns, employing GPR and ultrasonic methods as well as thermography.

Combining the GPR and UT methods is commonly used in imaging, monitoring and analyzing the condition of engineering structures. GPR is particularly useful to conduct surveys on large areas because it allows handling a large amount of data in a reasonable amount of time. In previous studies, the GPR method has shown high efficiency in the imaging of reinforcement bars [23,24], cracks [25], defects in the form of air voids, delamination and moisture [26,27,28] or systems applied for concrete strengthening [29,30]. On the other hand, the UT techniques were proved to work well in the detection of defects such as notches [31,32,33], micro- and macro-cracks [34], small air gaps [35] or minor scratches [36], as well as in the evaluation of plate-like structures [37,38] and adhesive materials connections [39,40]. What is more, ultrasounds can be applied for inspecting conductive materials, unlike electromagnetic waves used in the GPR method. If both methods are applied appropriately, they may be considered as complementary. The assessment of the examined element becomes more reliable by implementing these two measurement techniques. The literature shows that both methods ensure a unique way of imaging the studied surface, enabling thus to resolve multiple research problems. Guadagnuolo [41] juxtaposed the GPR and UT techniques while examining the walls and floors of a historical church. Binda et al. [42] implemented the UT and GPR research to verify the damages and possible preservation works of the walls and piers due to the restoration of a damaged cathedral. Furthermore, the parameters of the mortar used as a possible means of repairing a damaged wall were controlled by performing ultrasonic tests. Perez-Gracia et al. [43] combined the UT and GPR techniques in the assessment of the geometry and physical properties of historical columns. The above-mentioned papers have presented many successful applications of combined GPR and UT methods; however, a thorough comparison of these methods by analyzing measurement data recorded along the same traces is rather limited.

The paper presents the results of the integrated ultrasonic testing and ground penetrating radar inspection conducted in St. Nicholas’ Church in Gdańsk, Poland. The aim of the study was to present the practical aspects of the application of both techniques in detecting and imaging the underfloor inclusions, such as air voids, brick walls, pipes, rubble and human remains. Experimental measurements of the floor were conducted in the area of both (south and north) aisles, and also around a trial pit. Preliminary investigations were conducted on physical models consisting of two concrete slabs stacked on top of each other and gradually moved apart to simulate a slot of varying thickness, in order to better understand the phenomena of electromagnetic and ultrasonic wave propagation within the air voids and concentrated inclusions. In addition, the numerical simulations of electromagnetic waves were performed to support the interpretation of the GPR results. The analysis of the results obtained allowed concluding that GPR was suitable for the imaging of concentrated inclusions, whereas UT enabled detecting air voids. The presented research revealed the possibilities and limitations of both methods, indicating their complementarity in the context of non-destructive diagnostics of historical buildings.

## 2. Materials and Methods

### 2.1. Object of Investigations

Integrated GPR and ultrasonic inspection were conducted in St. Nicholas’ Church in Gdańsk, Poland (Google Maps coordinates in a WGS84 system: 54.352206 N, 18.651510 E). This historical object from the 14th century is the only church in the city, which survived World War II without destruction. However, due to the long process of the settlement of the pillars, some damages appeared and progressed in the structural elements. Recently, the process of damage has accelerated, resulting in the serious cracking of vaults, displacement of arches and deformation of the floor. The poor condition of the church led to its closure in November 2018 and undertaking of repair works.

**Figure 1:**
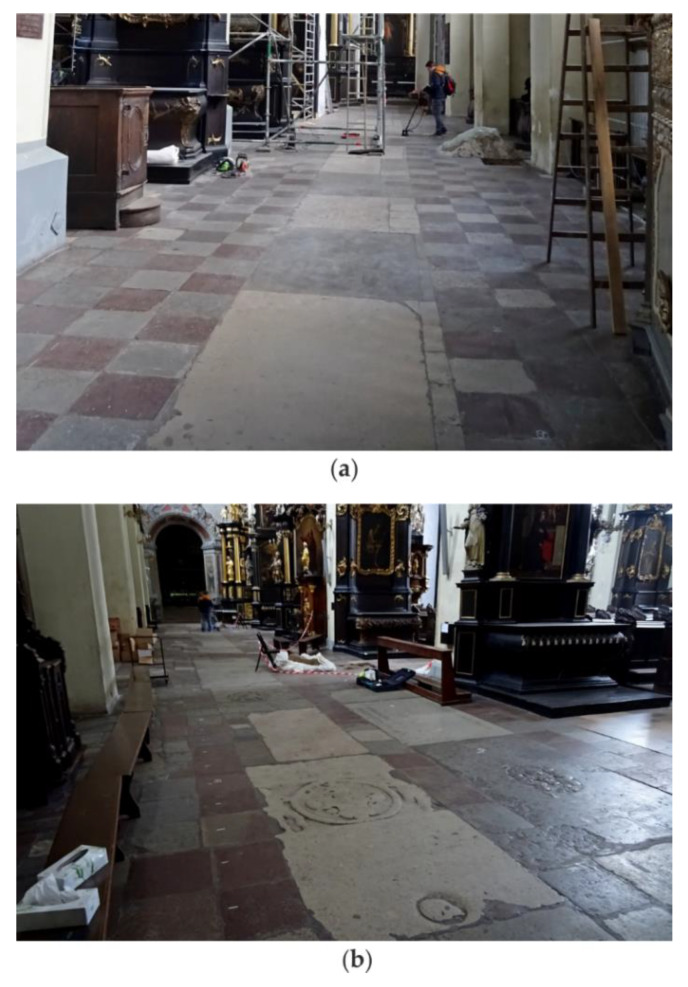
Photograph of the floor in St. Nicholas’ Church in Gdańsk, Poland (Google Maps coordinates in a WGS84 system: 54.352206 N, 18.651510 E): (**a**) south aisle; (**b**) north aisle.

**Figure 2:**
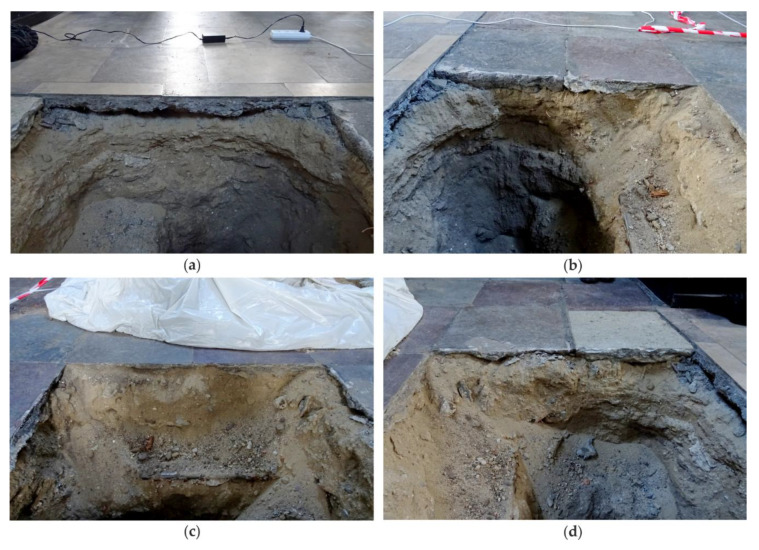
Photographs of the trial pit in the floor: (**a**) edge 1; (**b**) edge 2; (**c**) edge 3; (**d**) edge 4.

**Figure 3:**
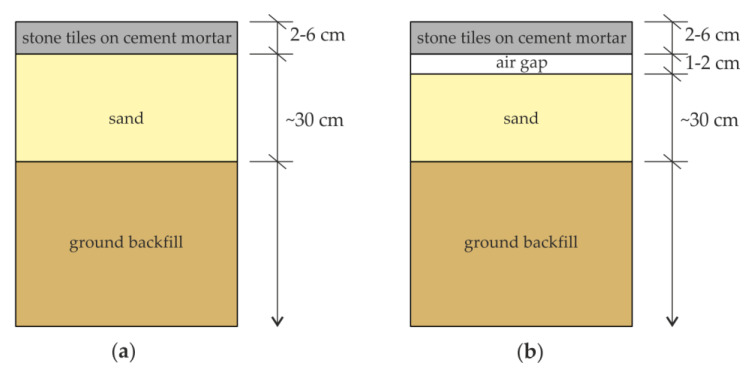
Schematic sketch of floor layers in the trial pit: (**a**) appropriate cross-section (without air gap); (**b**) cross-section with damage (air gap).

The conducted floor inspection reported in this paper was the first stage of investigations directed to assess the technical state of the church. NDT tests were performed on the floor of two aisles, south and north, as shown in Figure 1. In both aisles, the top floor layer was made of stone tiles measuring approximately 43 cm × 43 cm. In the central part of each aisle, tombstones were laid. The cross-section layers of the floor were identified in a few trial pits. One of the pits located in the north aisle is shown in Figure 2. Based on a visual inspection of the cross-section visible in the trial pit, particular layers in the floor were identified (Figure 3). It was found that the stone tiles laid on the cement mortar layer had a thickness of approximately 2–6 cm. Below was a layer of sand and ground backfill. It was also identified that at some edges, an air gap with a thickness of approximately 1–2 cm was visible below the cement mortar.

**Figure 4:**
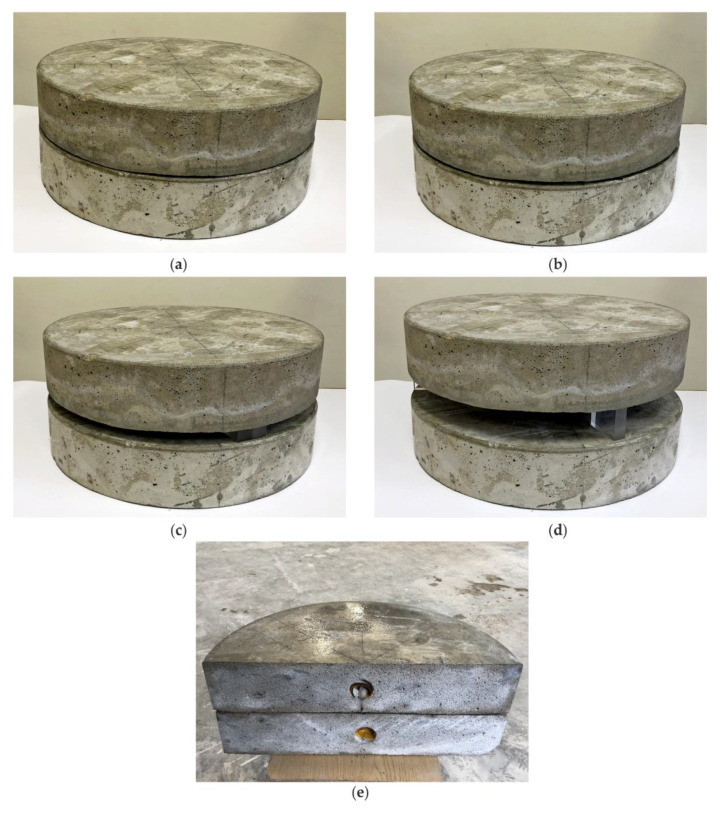
Circular concrete slabs with air gaps of different thickness: (**a**) 1 mm; (**b**) 4 mm; (**c**) 20 mm; (**d**) 50 mm; and (**e**) upper plate after cutting.

Additional investigations were conducted at the laboratory, on two circular concrete slabs. The slabs had a diameter of 49 cm and a thickness of 10 cm. One of the slabs had a table tennis ball with a diameter of 40 mm embedded in its center, at the half height of the slab. During the tests, the slabs were stacked on top of each other and gradually moved apart to simulate air gaps of varying thickness: 1, 4, 20 and 50 mm (see Figure 4).

### 2.2. Data Acquisition and Equipment

**Figure 5:**
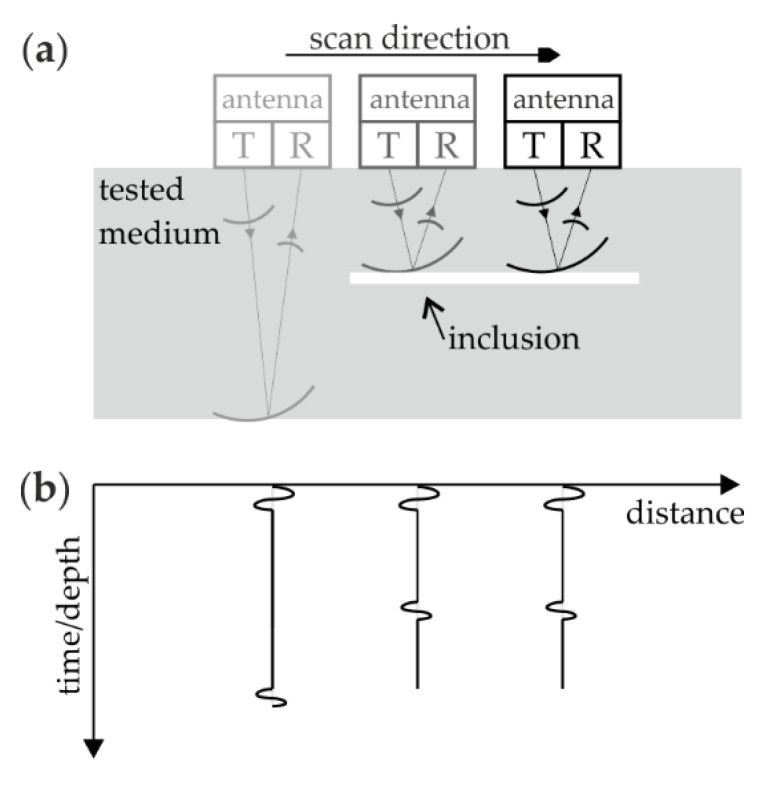
Scheme of measurements performed in pulse-echo mode: (**a**) collection of signals; (**b**) sketch of obtained echogram.

Two NDT techniques were used for inspecting the floor: ground penetrating radar and ultrasonic testing. In both approaches, a pulse-echo test system was used which comprises both the transmitting (T) and receiving (R) antennas. During the pulse-echo measurements (Figure 5), the antenna is moved along the tested surface and a single time signal (so-called an A-scan) is recorded for a specific position. The assembling of the A-scans gives an image called a B-scan or echogram. As the antenna approaches an element that differs in electrical (in the ground penetrating radar method) or mechanical (in the ultrasound method) properties from the surrounding medium, the time the wave returns to the receiving antenna changes. A reflection is then created on the echogram reflecting the disturbing element. A reflection from any point inclusion (e.g., circular air gap, reinforcing bar) is represented in the B-scan as a hyperbola, while longitudinal inclusions are represented as line patterns.

The Aladdin system (IDS GeoRadar, Pisa, Italy) equipped with a 2 GHz antenna was used to perform the GPR measurements. For each trace, the registered time range was 32 ns and the number of captured samples was 1024. The step distance between particular A-scans was 1 cm. The GPR data were registered in K2 FastWave and then processed in GRED HD by the following operations: automatic time zero correction, bandpass filtration in a frequency range of 500–3000 MHz and smoothed gain. Ultrasonic testing was performed using the pulse analyzer Pundit PL-200 (Proceq SA, Schwerzenbach, Switzerland) equipped with a 50 kHz antenna. For each A-scan, 1000 samples were captured with a time step of 1 µs, and the step distance between particular traces was 1 cm.

**Figure 6:**
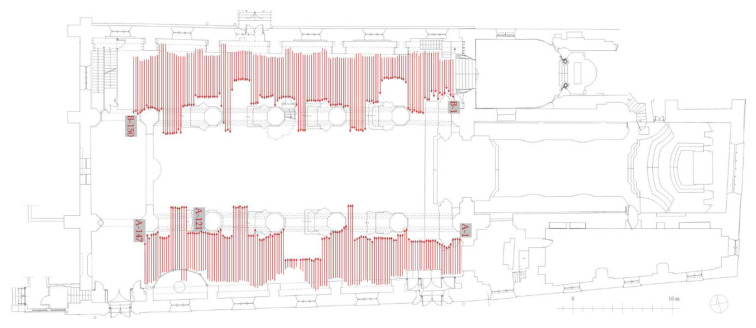
Plane view of the church with ground penetrating radar (GPR) traces in the south (traces A-1 to A-147) and north (traces B-1 to B-150) aisles.

**Figure 7:**
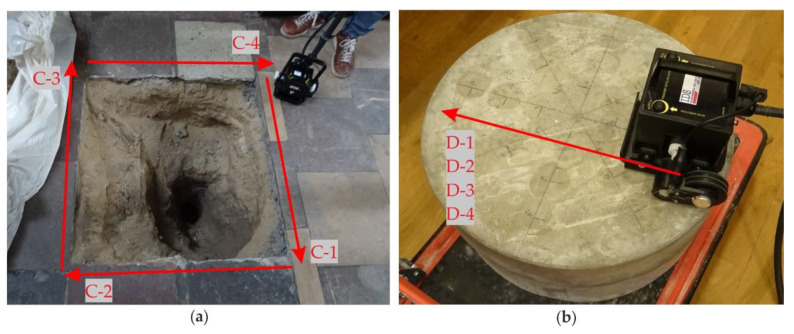
GPR traces registered along (**a**) edges of the trial pit (traces C-1 to C-4) and (**b**) the circular slabs with air gaps of varying thickness (traces D-1 to D-4).

**Figure 8:**
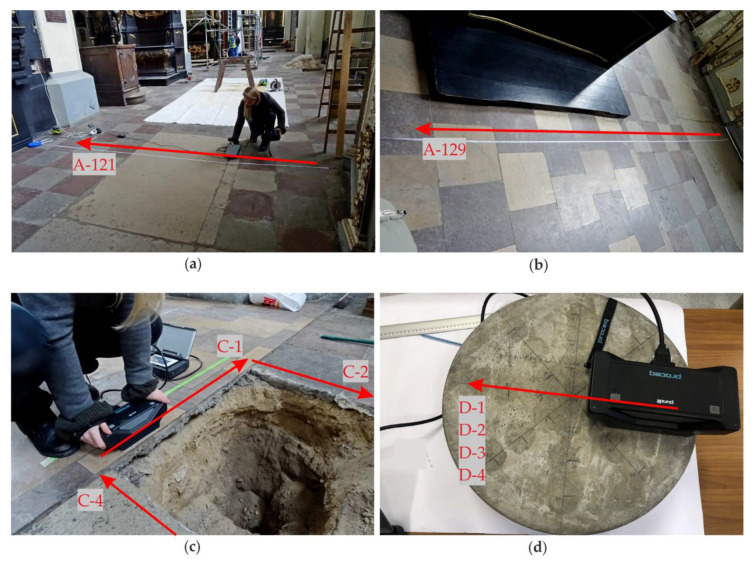
Ultrasonic measurements: (**a**,**b**) along traces A-121 and A-129 in the south aisle; (**c**) along edges of the trial pit; (**d**) along circular slabs.

The GPR inspection of the floor was conducted in both aisles (Figure 6). During the measurements, 147 profiles were traced in the south aisle (A-1 to A-147) and 150 profiles in the north aisle (B-1 to B-150). The distance between particular profiles was 21.5 cm. Some inaccessible areas were omitted during the GPR scanning, so the shape of the scanned area was irregular. Next, four scans were acquired along the edges of the trial pit (denoted as C-1, C-2, C-3 and C-4 in Figure 7a). Finally, one profile was traced on the concrete slabs. Four measurements were made, each for the different thicknesses of the air gap, i.e., D-1 (1 mm), D-2 (4 mm), D-3 (20 mm) and D-4 (50 mm). Additionally, along selected traces, UT measurements were carried out (see Figure 8). The summary of the conducted GPR and UT surveys is given in Table 1.

### 2.3. Numerical Simulations of Electomagnetic Wave Propagation

In order to better interpret the results obtained from the GPR studies, several numerical models with inclusions expected under the tested floor were prepared. The calculations with the models were performed to give information about the behavior of electromagnetic waves under the influence of anomalies, which can be used to analyze the GPR B-scans of the floor. Numerical modeling of electromagnetic wave propagation was carried out by the finite-difference time-domain (FDTD) method using the gprMax open source software (release 3.1.5) [44]. Two groups of 2D models were created. The first one (models #1) corresponded to the floor, while the second one (models #2) represented the concrete slab. The models were discretized using a 1 mm × 1 mm grid. The time step was selected automatically based on the Courant–Friedrichs–Lewy (CFL) condition. The outer space of the models was restricted by perfectly match layer (PML) absorbing boundary conditions. The excitation signal emitted by the transmitting antenna was the Ricker function with a central frequency of 2 GHz, and the distance between the transmitting and receiving antenna was set as 6 cm, according to the actual distance in the IDS antenna.

**Figure 9:**
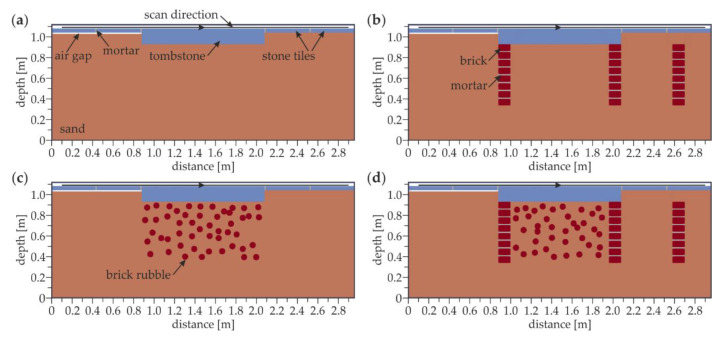
Finite-difference time-domain (FDTD) model of the floor: (**a**) model #1.1; (**b**) model #1.2; (**c**) model #1.3; (**d**) model #1.4.

The FDTD models of the floor are shown in Figure 9. The models with external dimensions of 2.96 m × 1.12 m were prepared in four variants. The aim of the simulations made on models #1.1–1.4 was to enhance the interpretation of the GPR surveys by analyzing how different a prior known underfloor inclusion influenced the registered echograms. Each model included four stone tiles with dimensions of 43 cm × 4 cm and a tombstone with the dimensions of 120 cm × 15 cm. An air gap with a thickness of 1 cm was inserted under two stone tiles (on the left side of the tombstone). The other two stones were laid directly on the ground (on the right side of the tombstone). Model #1.1 (Figure 9a) included a plain layer of sand under the stone tiles and tombstone. In model #1.2, three walls were inserted to represent underfloor crypts. The walls were made of bricks with dimensions of 6.5 cm × 12 cm and a 1 cm thick mortar. Additional two models (#1.3 and #1.4) comprised of concentrated inclusions in the form of brick rubble. The following values of the electric permittivity were adopted: ε*_r_* = 9 (tiles), ε*_r_* = 3 (sand), ε*_r_* = 6 (brick) and ε*_r_* = 4 (mortar). The conductivity for all materials was set as *σ* = 0.01 S/m. A-scans were registered at 280 nodes, starting from 0.08 m and giving the scan length of 2.79 m.

**Figure 10:**
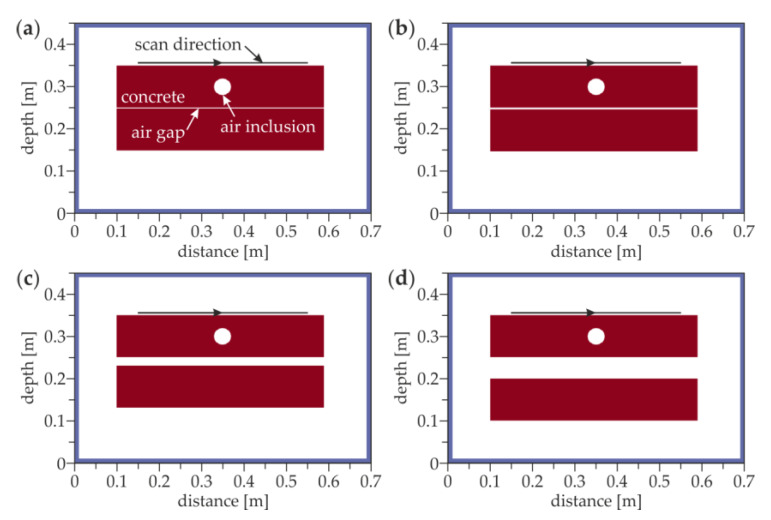
FDTD model of the concrete slabs with an air gap of the thicknesses (**a**) 1 mm (model #2.1); (**b**) 4 mm (model #2.2); (**c**) 20 mm (model #2.3); and (**d**) 50 mm (model #2.4).

Figure 10 illustrates the FDTD models of two concrete slabs separated with an air gap. The 2D models with external dimensions of 0.7 m × 0.45 m consisted of two concrete sections with dimensions of 0.49 m × 0.1 m separated by an air gap with a thickness of 1 (model #2.1), 4 (model #2.2), 20 (model #2.3) and 50 mm (model #2.4). In the upper slab, a circular air inclusion with a diameter of 40 mm was inserted. The electric permittivity of concrete ε*_r_* = 4, corresponding to the velocity of electromagnetic waves equal to 15 cm/ns, was determined using the “depth to known reflector” method [45]. The conductivity of concrete was adopted as *σ* = 0.01 S/m. During the FDTD simulations, 41 A-scans were registered, giving the scan length of 0.4 m.

## 3. Results and Discussion

### 3.1. Preliminary Investigations for Concrete Slabs

**Figure 11:**
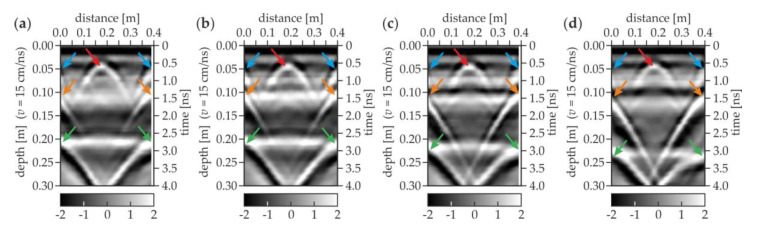
Experimental GPR B-scans for concrete slabs with air gap of thicknesses (**a**) 1 mm (trace D-1); (**b**) 4 mm (trace D-2); (**c**) 20 mm (trace D-3); and (**d**) 50 mm (trace D-4).

The results for the concrete slabs were first discussed as an initial step towards a more detailed analysis of the floor. Figure 11 shows the experimental GPR B-scans for the concrete slabs with different thicknesses of the air gap. The scan length was approximately 0.4 m. The depth axis was determined with the assumption that the electromagnetic wave velocity was equal to 15 cm/ns (based on the “depth to known reflector method” [45]). For the air gap thickness equal to 1 mm (Figure 11a), the half hyperbolas were clearly detected at the depth of 0.1 (orange arrows) and 0.2 m (green arrows), representing the reflections from the bottom faces of the upper and the lower slabs, respectively. This observation indicated the proper assumption of the electromagnetic wave velocity in the analyzed elements. The bottom faces of both slabs were visible as the lines with an intensity different from the adjacent part of the B-scan. The position of the reflection from the upper slab did not change, whereas the lower slab appeared deeper when increasing the air gap thickness (compare Figure 11a–d). For the air gaps with the thicknesses of 4, 20 and 50 mm, the position of the bottom plate was detected at about 20.5, 22 and 24 cm depths, respectively. This change showed the increase in the distance between both slabs and indicated a high compatibility of the obtained numerical results with the real thicknesses of the gaps. The identified gaps thicknesses were slightly different from the actual ones because the depth axis in Figure 11 was calculated for the velocity of the electromagnetic wave in concrete (15 cm/ns). In addition, the line at the depth of 0.1 m transformed into two separate lines, which indicated the opening of the air gap. It should also be mentioned that the line representing the bottom face of the lower slab became less pronounced for the greater distances between the slabs. This remark allowed concluding that the larger the air gap was, the harder it was to detect elements below it. Moreover, other half hyperbolas (denoted by blue arrows) were present at the depth of about 0.05 m, indicating a technological break during the concreting of the upper slab (visible in all B-scans, see Figure 11a–d). The upper slab was prepared in two stages unlike the lower one, in which no additional perturbations could be seen. A full hyperbola was observed in the center of each B-scan at the depth of about 0.05 m (marked by the red arrow), revealing the presence of the table tennis ball, which was placed in the upper slab between both stages of concreting.

**Figure 12:**
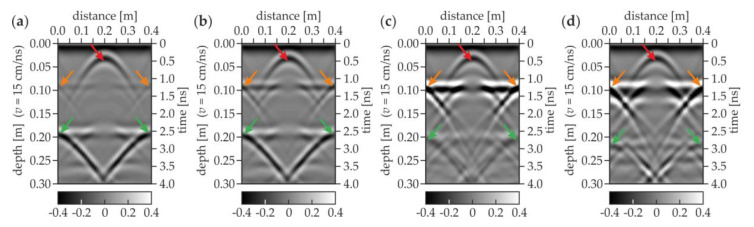
Numerical GPR B-scans for concrete slabs with air gap of thicknesses (**a**) 1 mm (model #2.1); (**b**) 4 mm (model #2.2); (**c**) 20 mm (model #2.3); and (**d**) 50 mm (model #2.4).

The numerical GPR B-scans corresponding with the above-described experimental results are presented in Figure 12. The half hyperbolas marked by the orange and green arrows clearly show the bottom faces of the upper and the lower slabs, respectively. The line denoting the bottom face of the lower slab became less noticeable and appeared deeper when increasing the distance between both slabs (cf. Figure 12a–d). For air gaps with thicknesses of 4, 20 and 50 mm, the position of the bottom plate is detected at about 19, 21 and 22 cm depths, respectively. The opening of the air gap was observed as the shift of the reflection from the bottom face of the lower slab occurred and also by the gradual separation of the single line into two lines. The hyperbola revealing the presence of the table tennis ball (red arrow) was clearly visible at the depth of 0.05 m. However, the technological break was not detectable (it was not modelled). The high agreement of the experimental and numerical results allowed concluding that the modelling of electromagnetic wave propagation was correct. The possibility of detecting air gaps and concentrated inclusions was confirmed.

**Figure 13:**
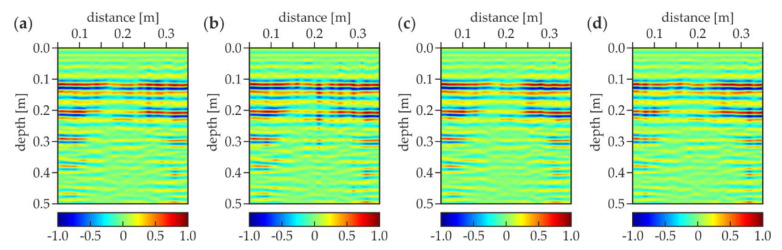
Ultrasonic B-scans for concrete slabs with air gap of thicknesses (**a**) 1 mm (trace D-1); (**b**) 4 mm (trace D-2); (**c**) 20 mm (trace D-3); and (**d**) 50 mm (trace D-4).

The UT B-scans for the analyzed slabs are presented in Figure 13. The ultrasonic pressure wave velocity determined before the main tests was equal to 2055 m/s. The scan length was reduced to 0.3 m due to the dimensions of the used UT antenna. There were no noticeable differences between all scans (Figure 13a–d), thus it could be concluded that the thickness of the air gap did not affect the results obtained. The lines at the depth of about 0.1 m representing the reflections from the bottom face of the upper slab were seen in all B-scans. Moreover, these lines were repeated regularly along the depth axis with the step of about 0.1 m (the slab thickness). Ultrasonic waves did not penetrate into the lower slab, they were fully reflected from the bottom face of the upper slab. The deeper lines were only the multiple reflections from the faces of the upper slab. The intensity of these lines decreased with the depth because of the damping of the ultrasonic waves. It is worth noting that the deeper lines appeared only near the edges of the slab. The presence of the boundaries strengthened the reflections, thus the signals were damped slower at the sides compared with the center of the slab. An important observation is that the table tennis ball was not observed at all. The reason might be the limitation of the used UT antenna. The Pundit PL-200PE instruction states that inclusions with a diameter of at least 30 mm should be detected, however, this condition deals with cylindrical elements. Being a relatively small spherical (concentrated) inclusion, the ball was not possible to be detected, despite the fact that its diameter was greater than minimum. To sum up, the air voids could be successfully detected using the UT technique, but without estimating the thickness. On the other hand, the content of the element below the air gap could not be imaged because the ultrasonic waves were entirely reflected from the air gap. Additionally, small concentrated inclusions could not be detected.

### 3.2. Numerical Models of the Floor

**Figure 14:**
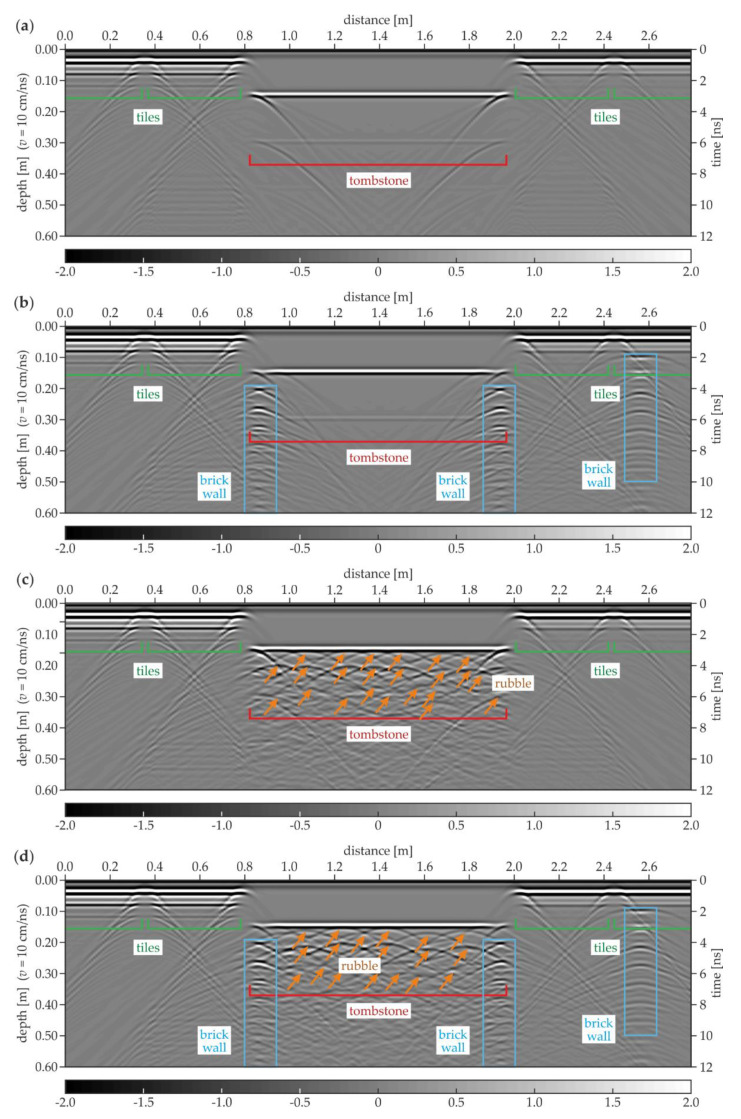
Numerical GPR B-scans for the floor: (**a**) model #1.1; (**b**) model #1.2; (**c**) model #1.3; (**d**) model #1.4.

Figure 14 shows the GPR B-scans acquired in the numerical models of the considered floor. Taking into account the complexity of the analyzed medium, the electromagnetic wave velocity was set as constant and equal to 10 cm/ns. The strategy applied did not take into account the heterogeneity factor. Therefore, the results were only approximations of the real subsurface geometry. However, this fact did not disable the proper interpretation of the results. In the model #1.1 (Figure 14a), the positions of the stone tiles were clearly identified based on the line patterns denoting the reflections from the vertical joints between tiles. The half hyperbolas at the ends of the tombstone were visible in the center of the scan. The lower faces of the tiles and the tombstone were located at the depths of 0.04 and 0.15 m, respectively. Some additional reflections with a lower intensity were observed below, indicating the further wave reflections from the analyzed elements (e.g., at the depth of 0.08 m below the tiles and 0.3 m below the tombstone). The difference between the tiles laid with and without the air gap was small, i.e., additional reflections below the tiles located above the air gap were insignificantly stronger. This observation might lead to the hypothesis that the detection of the small air gaps based on the GPR scanning could be problematic. For the model #1.2 (Figure 14b), the conclusions from the identification of the tombstone and the tiles were the same as for model #1.1. However, additional hyperbola patterns appeared, being the reflections from all singular bricks of the walls located below the edges of the tombstone. It is worth noting that the intensity of the hyperbolas decreased with the depth, which is a common relation observed for electromagnetic waves. Similarly, another brick wall was visible at the distance of 2.55 m. It is interesting to note that the walls seemed to be located at different depths. This apparent observation was the result of the assumption that the electromagnetic wave velocity was constant. The wave needed more time to travel though the tombstone (to reach the edge walls) compared with the stone tiles and the sand (above the additional wall). In the model #1.3 (Figure 14c), the additional hyperbolas were observed below the tombstone, proving the presence of the rubble. The great amount of the inclusions made the interpretation of the scan more complicated. It is also worth noting that the deeper hyperbolas had a much lower intensity, thus the identification of inclusions located deeper was straitened. The interpretation of the reflections below the tombstone was far more difficult when considering the simultaneous presence of the rubble and the brick walls (model #1.4, Figure 14d). The superimposition of the hyperbolas denoting the rubble and the singular bricks made them undistinguishable. Therefore, it was not possible to state whether a certain hyperbola denoted the brick or any different kind of inclusion. To sum up, the results obtained from the numerical calculations gave some information about the possibilities and limitations of GPR scanning. The location of the tombstone and the tiles could be determined. The rubble below the tombstone was detectable as well as the brick walls; however, when these elements appeared together, the interpretation was complicated. What is essential is that the air gaps were difficult for the identification.

### 3.3. Experimental Surveys for the Trial Pit

**Figure 15:**
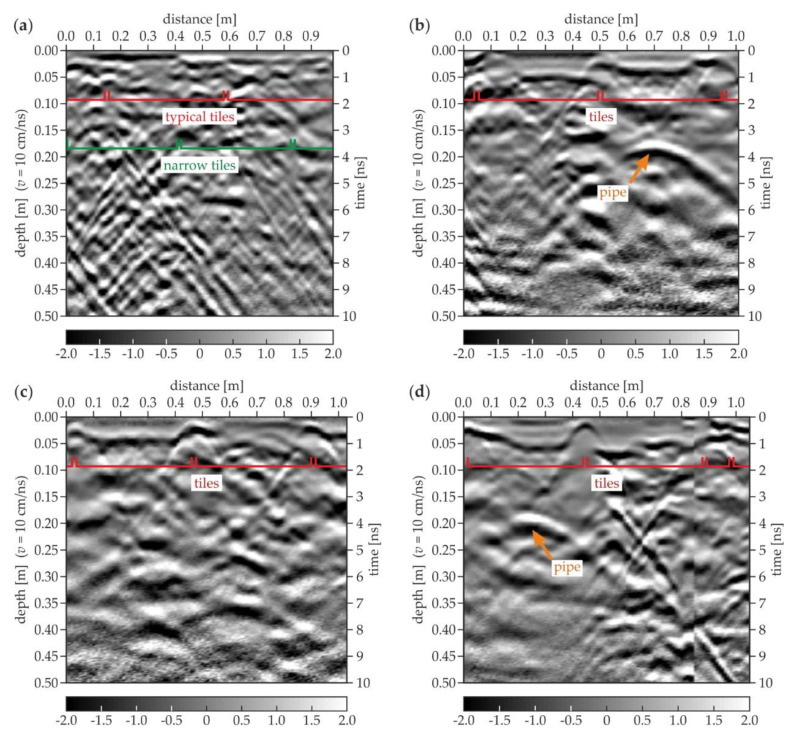
GPR B-scans for the trial pit: (**a**) edge 1 (trace C-1); (**b**) edge 2 (trace C-2); (**c**) edge 3 (trace C-3); (**d**) edge 4 (trace C-4).

Figure 15 presents the GPR B-scans collected around the trial pit. As can be seen in Figure 7a, several rectangular tiles formed a joint at one of the pit’s sides. Therefore, during the acquisition of the C-1 scan (Figure 15a), the GPR antenna was moved along the joint, thus the bottom faces of the tiles were not clearly imaged. However, the analysis of the upper part of the scan allowed identifying two different rows of tiles; the typical tile (43 cm × 43 cm) row was replaced at the edge of the trial pit with a narrower one (43 cm × 10 cm) laid with the overlap (cf. Figure 2a). The overlap length was about 0.16 m and it could be seen the clearest at the distance of 0.42–0.58 m. The ground under the C-1 scan had many inclusions, resulting in numerous irregularly distributed reflections (cf. the concentrated inclusions in Figure 14c,d). The C-2 scan (Figure 15b) clearly presents the line patterns being the reflections from the joints. The bottom faces of the tiles were identified at dissimilar levels, therefore the tiles (or the mortar layers below them) had different thicknesses. The hyperbola located at the distance of 0.7 m and the depth of 0.18 m denoted the presence of a metallic (steel or aluminum) pipe (cf. Figure 2). The ground on the right side of the scan (around the pipe, at the distance of 0.5–1.0 m) seemed less heterogeneous than the remaining part. This might be caused by the fact that the original ground was removed and replaced with another, more homogeneous one during placing the pipe under the floor. The C-3 scan (Figure 15c) shows the clear image of two tiles, one with the regular bottom face and the second with an unbalanced shape. The ground seemed to be original because it is highly heterogeneous, such as in the C-1 scan. The C-4 scan (Figure 15d) also clearly shows two typical tiles together with the narrow one (located at the distance of 0.88–0.98 m). The shape of the bottom face of all tiles was irregular. The shift was observed in the scan (at the distance of 0.85 m), caused by the slip of the antenna at the joint between the standard and the narrow tile (cf. Figure 2d). The reflection from the pipe was visible at the distance of 0.25 m and the depth of 0.18 m (the same as observed in the C-2 scan). The ground around the pipe was less inhomogeneous (the distance of 0.0–0.5 m) compared with the remaining part. Generally speaking, it was difficult to say whether there were any air gaps under the floor. The subtle difference between the image of the tiles with and without the air gap (observed in the numerical results) was here blurred by the ground inhomogeneities and a signal noise.

**Figure 16:**
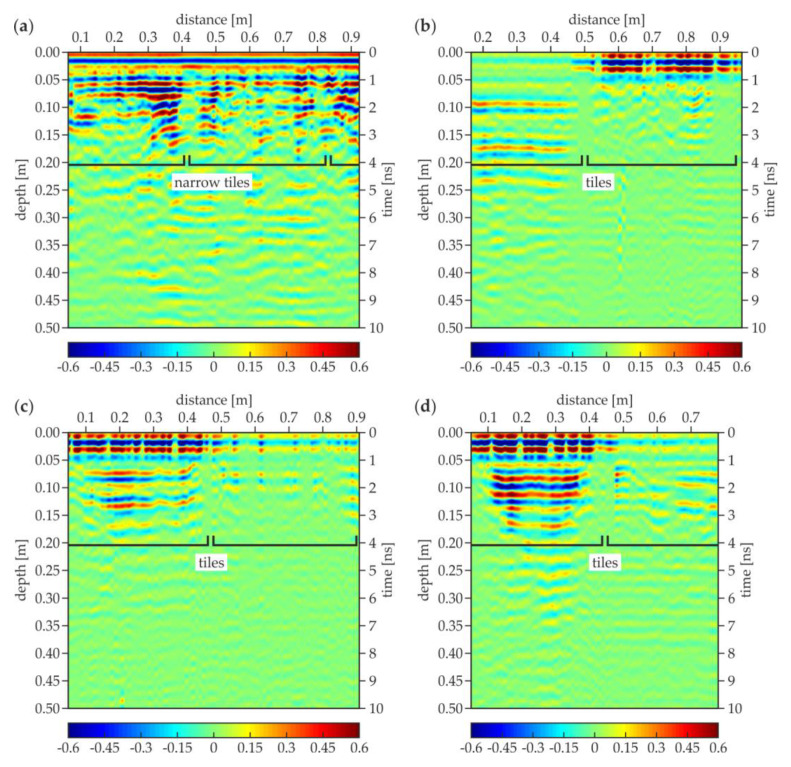
Ultrasonic B-scans for the trial pit: (**a**) edge 1 (trace C-1); (**b**) edge 2 (trace C-2); (**c**) edge 3 (trace C-3); (**d**) edge 4 (trace C-4).

Figure 16 contains the UT B-scans corresponding to the above-discussed GPR B-scans. The C-1 scan (Figure 16a) was performed through the narrow tiles, as can be seen in Figure 7a, thus the joints of the standard tiles were not observed. In the top part of the scan (at the depth of about 0.03 m), there was a straight line denoting the regular bottom face of the narrow tiles. However, there was no regularity in the lower part of the scan, where many multiple reflections occurred. Compared with the results for the concrete slabs, it might be stated that the air gap with an irregular shape was present in this area. The non-uniform settlement of the original ground probably led to the appearance of the air gap. The C-2 scan (Figure 16b) shows the image of two different tiles. The right tile, unlike the left one, was clearly visible at the top of the scan; it was possibly removed and then placed again with the use of a different (stronger) mortar. The multiple reflections appeared under the left tile, indicating the presence of the air gap (this tile laid on the original ground). On the contrary, there were no reflections under the right tile. The pipe detected in the GPR scans was not visible, which stays in agreement with the results for the concrete slabs where the UT scans did not reveal the table tennis ball. This observation allowed concluding that concentrated inclusions could not be detected by the UT scanning. It also should be mentioned that, according to the UT antenna instruction, the diameter of the pipe did not exceed 30 mm. In the C-3 scan (Figure 16c), two tiles were also visible. The left one was much more pronounced: it could have been replaced during some renovation works. Additionally, there were multiple reflections under both tiles, indicating the presence of the air gaps (resulting from the ground settlement). However, it needs to be noted that the reflections were less intensive under the right tile because it was weakly identified itself. The possibility of detecting the tiles in the C-4 scan (Figure 16d) was also distinguished: the left one was much better imaged. Although the ground below the left tile was replaced, the multiple reflections indicating the presence of an air gap were visible under both tiles. Like in the C-3 scan, the reflections under the right tile were weaker because it was weakly imaged itself. It also needs to be added that the pipe was not observed like in the C-2 scan.

### 3.4. Experimental Surveys for Floor

**Figure 17:**
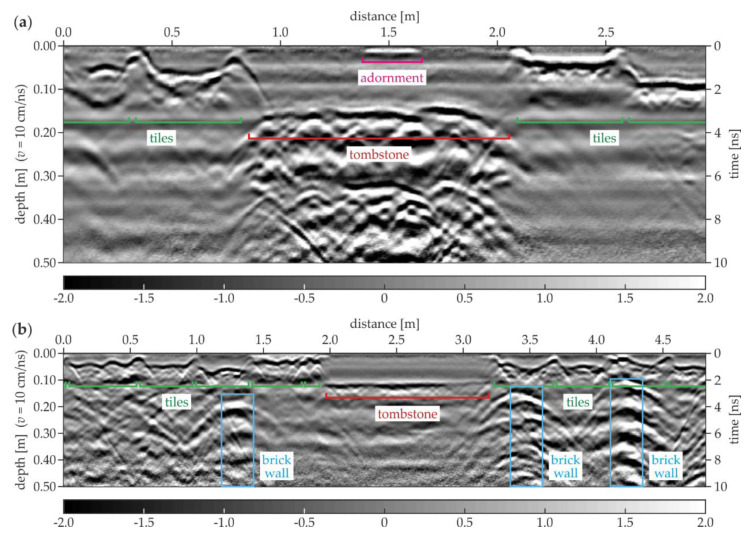
GPR B-scans for the south aisle (survey A): (**a**) A-19; (**b**) A-117.

Figure 17 presents two examples of the GPR B-scans acquired in the area of the south aisle. The A-19 scan (Figure 17a) clearly reveals the location of the tiles based on the reflections from the tile grouts. The bottom faces of the tiles were identified at different depths, thus they had different thicknesses, varying between 0.04 and 0.09 m. The tombstone with a thickness of approximately 0.16 m could be observed in the center of the scan. An additional reflection denoting the tombstone adornment was also visible at the top of the scan. The ground under the tombstone had many concentrated inclusions, probably being the rubble or the human remains from the original crypts (cf. Figure 14c,d). Aside from this area, inhomogeneities were not observed. It was difficult to state whether there were any brick walls under the edges of the tombstone because the hyperbolas denoting the concentrated inclusions blurred the image. The A-117 scan (Figure 17b) shows the tiles with different thicknesses between 0.03 and 0.08 m. The tombstone was also visible; however, its shape was far more regular compared with the one from the A-19 scan. The ground below the tombstone had no strong inhomogeneities, thus it could be stated that not all the tombstones were laid in the area of the original crypts. Other interesting reflections were the hyperbolas located vertically one above another at the distances of 1.3, 3.4 and 4.2 m. They indicated the presence of three brick walls (cf. Figure 14b,d), that might be the remainders of the original supporting structures of the crypts.

**Figure 18:**
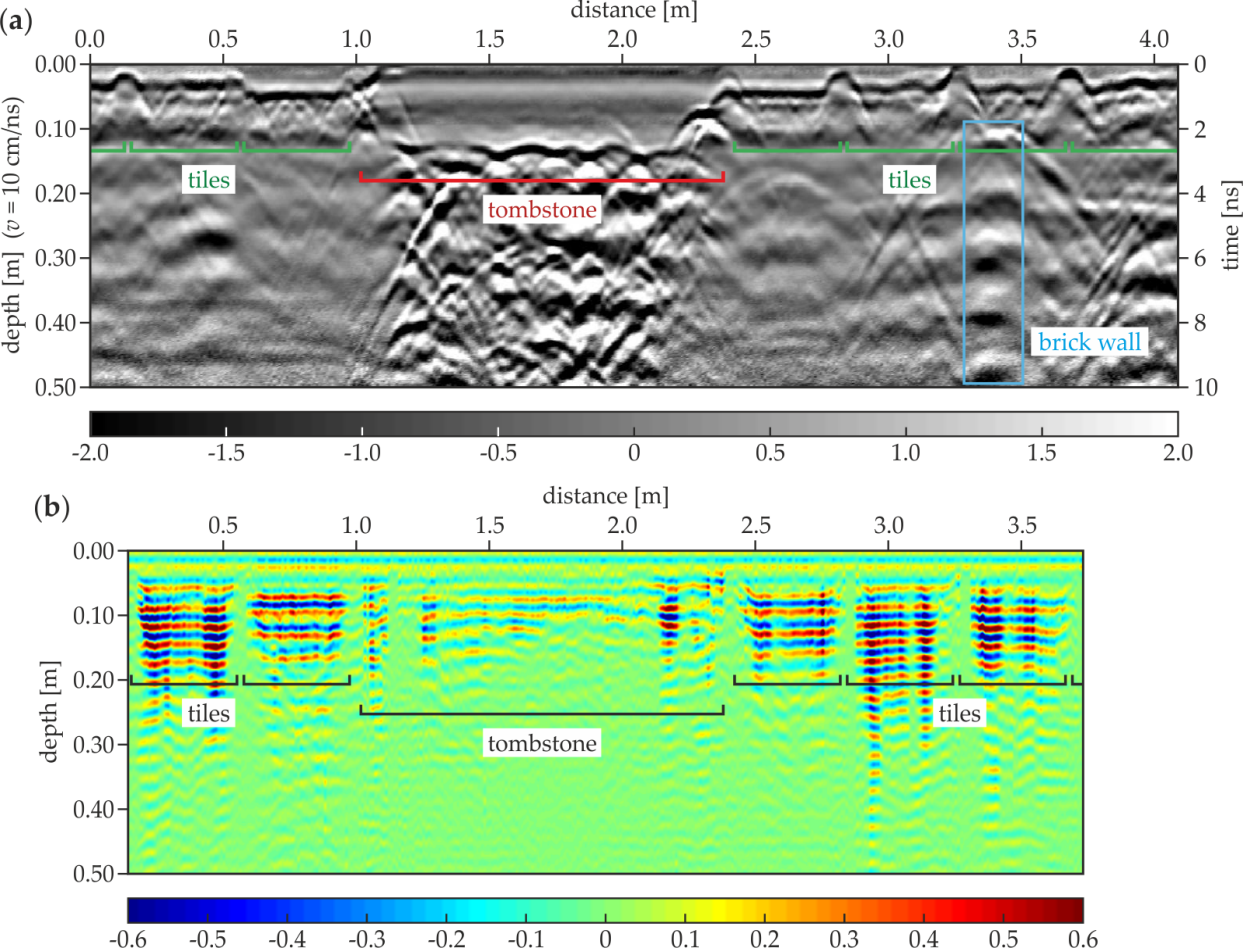
GPR and UT B-scans for trace A-121 (south aisle): (**a**) GPR scan; (**b**) UT scan.

**Figure 19:**
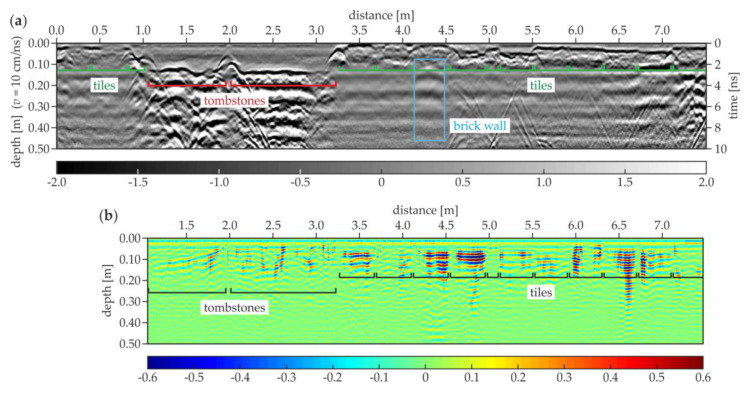
GPR and UT B-scans for trace A-129 (south aisle): (**a**) GPR scan; (**b**) UT scan.

The next two GPR B-scans from the south aisle are presented together with the corresponding results of the UT scanning. In the A-121 GPR B-scan (Figure 18a), the tiles with different thicknesses (in the range of 0.02–0.04 m) were visible, and also a tombstone with the thickness of about 0.12 m was identified. The ground below the tombstone contained some concentrated inclusions (probably rubble or human remains). The brick wall was present at a distance of about 3.4 m. The UT scan (Figure 18b) clearly revealed the tile pattern, and also the tombstone was observed. The presence of the multiple reflections under each tile allowed stating that there were many air gaps below them, which agreed with the fact that the floor of the south aisle experienced significant settlement. The reflections denoting the air gaps generally did not appear below the tombstone, probably because of its weight. What is also worth noting is that no additional reflections from the ground heterogeneities could be identified. The UT scanning did not detect concentrated inclusions, which were already observed for the pipe in the area of the trial pit and the table tennis ball in the concrete slab. The GPR B-scan for the trace A-129 is presented in Figure 19a. The tiles with different thicknesses (varying between 0.02 and 0.06 m) are clearly detectable. Two tombstones with a thickness of approximately 0.12 m and irregular shape of the bottom face were also present. The ground under both tombstones had many concentrated inclusions unlike aside from this area. The brick wall could be visible at the distance of about 4.3 m; however, the intensity of the reflections from the bricks was not as strong as in the A-121 scan. In the UT scan (Figure 19b), the tiles and the tombstones could be localized. There were no significant reflections under the tombstones, thus the air gaps were not likely to be there, unlike under the most of the tiles, where multiple reflections appeared. This observation corresponded with the settlement of the floor in the area of the south aisle.

**Figure 20:**
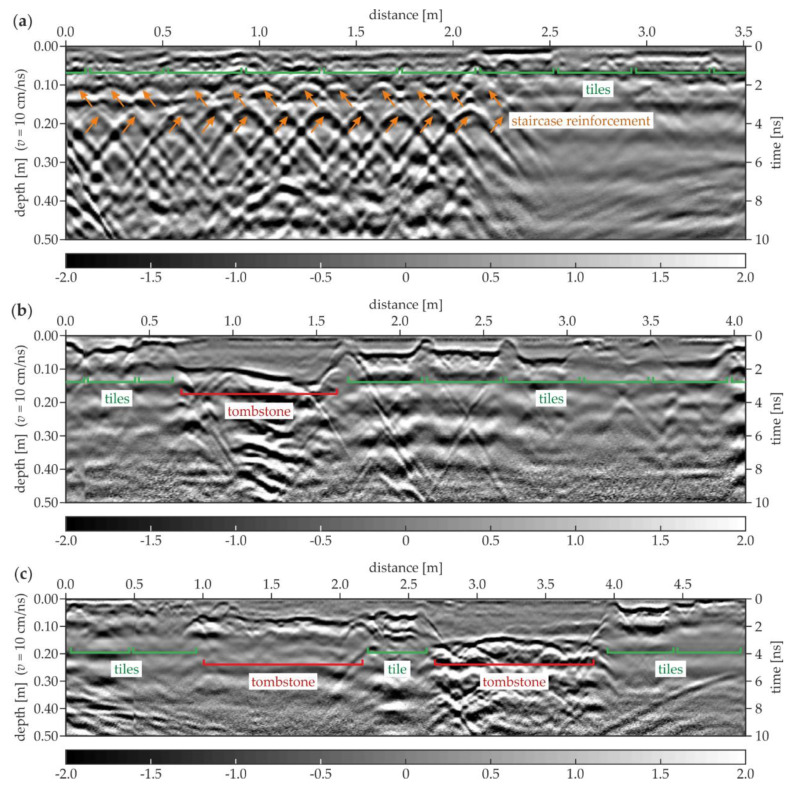
GPR B-scans for the north aisle (survey B): (**a**) B-5; (**b**) B-35; (**c**) B-143.

Figure 20 shows the examples of the GPR B-scans for the north aisle. The B-5 scan (Figure 20a) shows the regular pattern of tiles with a constant thickness of 0.03 m. Several evenly spaced hyperbolas were visible at two levels (the depth of 0.11 and 0.18 m) and the distance of 0.0–2.1 m. These reflections represented the reinforcement of the staircase located under the floor. The ground below the tiles aside of the staircase seemed to be devoid of significant heterogeneity. In the B-35 scan (Figure 20b), the tiles had different thicknesses in the range of 0.02–0.06 m. The tombstone had a thickness varying between 0.10 and 0.15 m and the skew shape of the bottom face. The ground below the tombstone had some concentrated inclusions. The B-143 scan (Figure 20c) presents the tiles with the thicknesses between 0.02 and 0.05 m. Two tombstones were also visible, the left one with the thickness of about 0.09 m and the right with the thickness of approximately 0.16 m. The ground under the thinner tombstone did not have inhomogeneities, unlike the ground under the second one, where multiple reflections from the concentrated inclusions occurred. This observation confirms the fact that some of the tombstones were placed aside from the area of the original crypts.

### 3.5. GPR Tomographic Imaging of Floor

**Figure 21:**
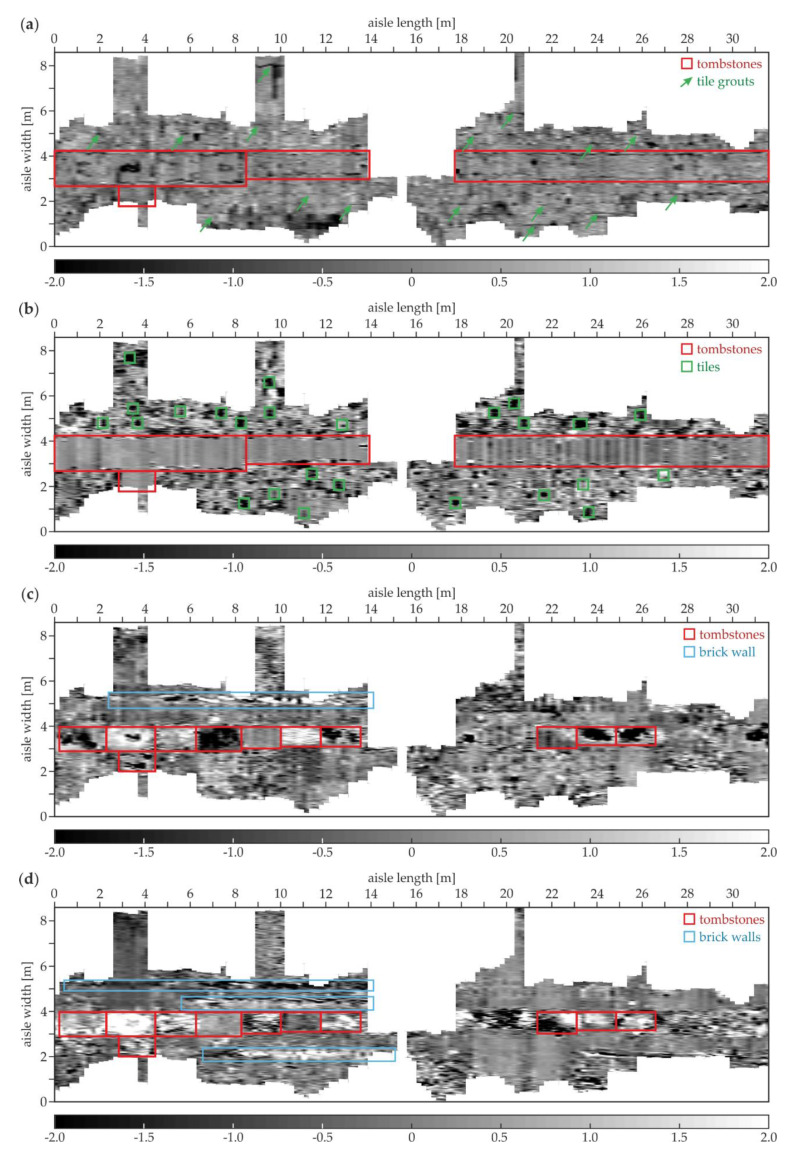
GPR tomographic images of the south aisle (survey A) at different depths: (**a**) 0.16 cm; (**b**) 4.06 cm; (**c**) 12.19 cm; (**d**) 23.75 cm.

Figure 21 presents the tomographic images of the south aisle. At the depth of 0.16 m (Figure 21a), the tombstones identified in the above-discussed B-scans of the south aisle were detected. The elements were laid in a straight line along the whole aisle at the width between about 2.75 and 4.25 m. Additionally, the single tombstone was located aside from the main row at the length between 2.5 and 4.5 m (it was seen in the A-129 scan, Figure 19). The distinction of singular tombstones from the main row was not possible; the scanning was performed along the tombstone grouts, thus they could not be imaged. The singular tiles were not detected due to a single direction profiling. The tile grouts in the lengthwise direction (perpendicular to the measurement traces) were identified, unlike the crosswise grouts (oriented in the same direction as the scan traces). At the depth of 4.06 cm (Figure 21b), the imaging of the tombstones was far clearer; however, there was still no possibility to distinguish the singular elements. On the contrary, the individual tiles could be detected; the chosen tomography depth was between the minimum and the maximum thickness of the tiles, thus some of them were visible, whereas other ones could not be observed. At the depth of 12.19 cm (Figure 21c), most of the tombstones were clearly visible. The lower faces of some thicker tombstones were below the tomography depth, unlike the thinner ones, which were not detected at this level. The adornment was detected on the second tombstone from the left. The tiles could not be identified because their bottom faces were located certainly above the actual tomography level. What is more, the brick wall was moderately imaged at the width of about 5.2 m and the length between 2.2 and 13.8 m. The tombstones were still distinguishable at the depth of 23.75 cm (Figure 21d). The inhomogeneities in the ground under certain tombstones were visible, especially on the right side of the tomography. The brick wall discovered at the depth of 12.19 cm was still detectable and seemed to be longer. What is more, another two brick walls were identified: the first one at the width of 4.5 m and the length between 5.5 and 14.0 m, the second at the width of 2.2 m and the length between 6.5 and 15.0 m. All of the three walls were clearly imaged in the A-117 B-scan (see Figure 17b).

**Figure 22:**
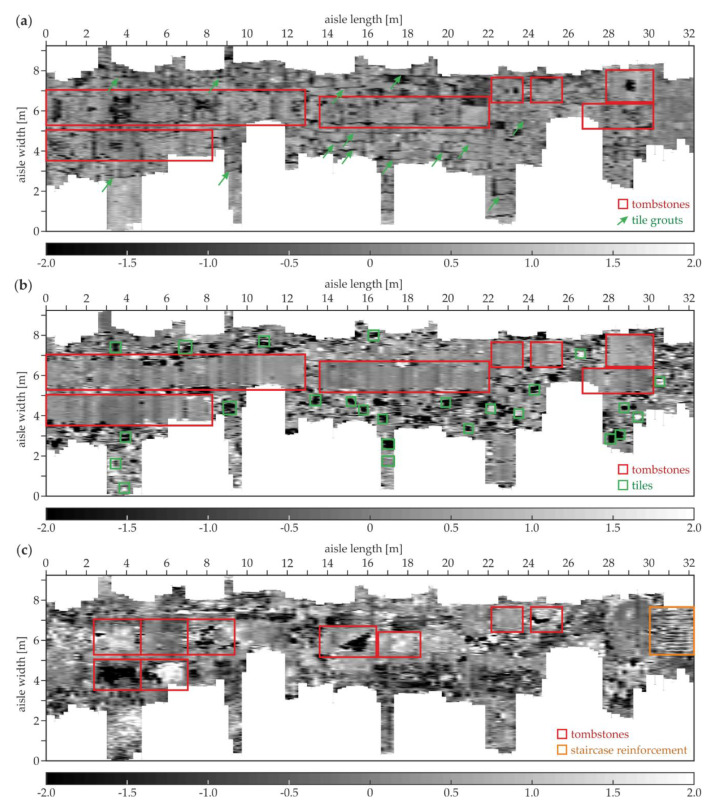
GPR tomographic images of the north aisle (survey B) at different depths: (**a**) 0.16 cm; (**b**) 4.06 cm; (**c**) 12.19 cm.

Figure 22 shows the tomographic images of the north aisle. Similarly to the results for the south aisle, the tombstones were detected at the depth of 0.16 cm (Figure 22a); however, their configuration was not so regular. The lengthwise tile grouts were also imaged, unlike the crosswise grouts. The adornments were visible on some of the tombstones. At the depth of 4.06 cm, the tombstones were shown far more clearly. Some individual tiles were also observed. At the depth of 12.19 cm, the singular tombstones could be distinguished. Some of them did not seem to be rectangular because of the irregular shape of their bottom faces. It is also worth noting that the staircase reinforcement was visible at the length of 30.0–32.0 m and the width of 5.2–7.8 m. Only the lengthwise bars were noticeable, and the crosswise reinforcement could not be imaged because of the single direction of the GPR scanning.

## 4. Conclusions

In this study, the integrated GPR and UT inspection was conducted on the floor of a historical church. The performed investigations focused on the detection of air gaps and other anomalies located under the stone tiles and the tombstones. The research was supported by laboratory models illustrating the behavior of electromagnetic and ultrasonic waves. The numerical models were prepared to illustrate the propagation of electromagnetic waves in the medium containing inclusions such as air gaps, brick walls, pipes and brick rubble. The results obtained provided useful information about the possibilities and limitations of the GPR and UT methods.

The possibility of an efficient application of the GPR technique for detecting small concentrated inclusions was confirmed. The table tennis ball was identified in the concrete slab (both in the numerical and experimental results); the pipe was detected in the area of the trial pit; some bricks and concentrated inclusions were observed in the ground under the floor. The larger surface and volume elements (stone tiles, tombstones) were successfully imaged too. The GPR method was also able to show the air gaps as lines (single or double, depending on the air gap thickness). However, the exact imaging of the air voids was possible only for thick layers; in the case of thin air gaps, it was difficult to state whether the lines denoted the air gap or a boundary between two media.

The UT measurements allowed an efficient detecting of the air gaps, independent of their thickness. This resulted from the phenomenon of total reflection of elastic waves at the boundary of the analyzed element. The location of stone tiles and tombstones could be also clearly visible. On the other hand, because of wave reflection, the UT inspection did not allow to detect anything below the air gap. What is more, ultrasonic waves did not identify concentrated inclusions, such as the table tennis ball, the pipe and the ground inhomogeneities, which could be caused by the limitations of the UT antenna used.

The tomographic snapshots provided the overall image of the examined area at a specific depth. This type of imaging could be useful when the exact location of underfloor inclusions in relation to the entire scanned area is the object of interest. However, when the detailed profile of a part of considered structure is important, an analysis of B-scans is more appropriate. For the presented study, a good compliance of both ways of imaging was observed.

To increase the applicability and practicality of non-destructive inspection techniques, the use of integrated GPR and UT methods was recommended. The methods complemented each other, allowing an exclusion of their limitations. The UT technique was efficient at visualizing air gaps of different thicknesses, however, it was not suitable for imaging small inclusions and anything below the air voids. What is also important, the UT measurements were time-consuming. The GPR method successfully detected concentrated inclusions with different sizes; it also allowed inspecting a large area with a relatively low time cost. However, the GPR measurements did not allow detecting air voids.

To summarize, the integrated inspection combining the GPR and UT techniques appeared to be effective for non-destructive diagnostics of underfloor structures in cultural heritage buildings. The proposed approach can be useful for the detection of anomalies laying under the floor, such as air gaps, bricks and pipes, which can appear in historical objects. The complementarity of both methods enables a precise analysis of the tested structure.

## Figures and Tables

**Table 1:** Scheme of GPR and ultrasonic testing (UT) investigations.

Surveys	GPR Traces	UT Traces
A	A-1 to A-147	A-121 and A-129
B	B-1 to B-150	−
C	C-1 to C-4	C-1 to C-4
D	D-1 to D-4	D-1 to D-4
